# Insights on the marine microbial nitrogen cycle from isotopic approaches to nitrification

**DOI:** 10.3389/fmicb.2012.00356

**Published:** 2012-10-12

**Authors:** Karen L. Casciotti, Carolyn Buchwald

**Affiliations:** ^1^Department of Environmental Earth System Science, Stanford UniversityStanford, CA, USA; ^2^MIT/WHOI Joint Program in Chemical OceanographyWoods Hole, MA, USA

**Keywords:** nitrification, isotopic fractionation, oxygen, nitrogen, nitrate, nitrous oxide

## Abstract

The microbial nitrogen (N) cycle involves a variety of redox processes that control the availability and speciation of N in the environment and that are involved with the production of nitrous oxide (N_2_O), a climatically important greenhouse gas. Isotopic measurements of ammonium (NH^+^_4_), nitrite (NO^−^_2_), nitrate (NO^−^_3_), and N_2_O can now be used to track the cycling of these compounds and to infer their sources and sinks, which has lead to new and exciting discoveries. For example, dual isotope measurements of NO^−^_3_ and NO^−^_2_ have shown that there is NO^−^_3_ regeneration in the ocean's euphotic zone, as well as in and around oxygen deficient zones (ODZs), indicating that nitrification may play more roles in the ocean's N cycle than generally thought. Likewise, the inverse isotope effect associated with NO^−^_2_ oxidation yields unique information about the role of this process in NO^−^_2_ cycling in the primary and secondary NO^−^_2_ maxima. Finally, isotopic measurements of N_2_O in the ocean are indicative of an important role for nitrification in its production. These interpretations rely on knowledge of the isotope effects for the underlying microbial processes, in particular ammonia oxidation and nitrite oxidation. Here we review the isotope effects involved with the nitrification process and the insights provided by this information, then provide a prospectus for future work in this area.

## Nitrification in the ocean—roles in NO^−^_3_ supply and N_2_O production

Nitrification comprises a key link in the marine nitrogen (N) cycle converting the most reduced form of N (ammonia, NH_3_) to the most oxidized (nitrate, NO^−^_3_). Although sunlight appears to partly inhibit nitrification (Olson, 1981a; Guerrero and Jones, 1996; Merbt et al., 2012), there are many indications that nitrification occurs in the euphotic zone (Ward, 1985, 2005; Wankel et al., 2007; Yool et al., 2007; Clark et al., 2008). Therefore, when reduced organic N is released into solution through cell lysis, grazing and digestion, it can be either reassimilated or oxidized back to NO^−^_3_ in the sunlit surface waters. Also, when particulate organic matter (in the form of detritus, fecal pellets, or marine snow) sinks out of the euphotic zone, it is gradually broken down into its component parts and remineralized into its inorganic forms: CO_2_, NH^+^_4_, and PO^3−^_4_. In oxic water columns, the NH^+^_4_ released from organic matter remineralization below the euphotic zone is rapidly oxidized to NO^−^_3_. The distribution of nitrification rates in the ocean is therefore expected to follow the distribution of NH^+^_4_ supply from organic matter remineralization, which decreases exponentially with depth (Ward and Zafiriou, 1988).

Nitrification is carried out through the combination of two microbial processes: ammonia oxidation to NO^−^_2_ and nitrite oxidation to NO^−^_3_. Ammonia oxidation is a chemoautotrophic process carried out by ammonia-oxidizing bacteria (AOB) and ammonia-oxidizing archaea (AOA). These organisms use NH_3_ as their source of reducing power for CO_2_ fixation and energy production. Nitrite oxidation is also a chemoautotrophic process and is carried out by nitrite-oxidizing bacteria (NOB). These bacteria use nitrite (NO^−^_2_) as their source of reducing power for CO_2_ fixation and energy production (Watson, 1965; Bock et al., 1989). Most ammonia and nitrite oxidizers are obligate chemoautotrophs (Watson and Waterbury, 1971), although a few are able to grow mixotrophically (Watson et al., 1986).

Although NO^−^_2_ is an intermediate in the nitrification process, it rarely accumulates in the ocean. NO^−^_2_ can be found at the base of the euphotic zone in a feature termed the primary nitrite maximum (PNM; Wada and Hattori, 1971). The processes contributing to NO^−^_2_ accumulation in the PNM are still debated, but most likely include a combination of ammonia oxidation and nitrite oxidation, as well as assimilatory nitrate and nitrite reduction by phytoplankton (Ward et al., 1982, 1989; Dore and Karl, 1996; Lomas and Lipschultz, 2006; Mackey et al., 2011). The relative contributions of these processes to NO^−^_2_ cycling have different implications for N biogeochemistry and the links between C and N cycling. Net production of NO^−^_2_ through nitrification (decoupling of ammonia and nitrite oxidation) can also have implications for the production of nitrous oxide (N_2_O), a climatically important greenhouse gas. It is therefore important to know how the processes contributing to the production and maintenance of the PNM vary in space and time.

NO^−^_2_ also accumulates in oxygen deficient regions of the water column in a feature termed the secondary nitrite maximum (SNM; Brandhorst, 1959). The SNM is generally assumed to reflect active denitrification in oxygen deficient zones (ODZs), as SNM features are only found in the absence of dissolved oxygen (Brandhorst, 1959; Cline and Richards, 1972; Codispoti and Christensen, 1985). However, recent studies have shown that the presence of a SNM feature may not coincide with the most intense NO^−^_2_ cycling, as active NO^−^_2_ reduction occurs in the Omani upwelling region in the absence of NO^−^_2_ accumulation (Jensen et al., 2011; Lam et al., 2011). NO^−^_2_ consumption in the SNM may occur through many processes, including denitrification (reduction of NO^−^_2_ to N_2_), anaerobic ammonia oxidation (reduction of NO^−^_2_ to N_2_ and oxidation to NO^−^_3_), and nitrite oxidation (oxidation of NO^−^_2_ to NO^−^_3_). Recent studies using natural abundance isotopes (Casciotti, 2009), profile modeling (Lam et al., 2011), isotope tracers (Lipschultz et al., 1990; Füssel et al., 2012), and gene markers (Füssel et al., 2012) suggest that a significant fraction of NO^−^_2_ produced within the SNM may be consumed through oxidation to NO^−^_3_.

Several questions remain about the roles of AOB and AOA in marine nitrification, the controls on their distribution and activity, and the rates of these processes. These questions relate to the cycling of NO^−^_3_, NO^−^_2_, and NH^+^_4_ in the water column, and the production of N_2_O linked to nitrification. These questions can be addressed with a variety of complementary approaches, including molecular community analysis and quantification, instantaneous rate measurements, natural abundance stable isotope measurements, and geochemical modeling.

Examples of applications involving the use of natural abundance stable isotopes to study nitrification include: (1) the role of euphotic zone nitrification in supplying NO^−^_3_ for photosynthetic growth (Wankel et al., 2007; DiFiore et al., 2009), (2) the contributions of nitrification and nitrate reduction to NO^−^_2_ accumulation in the PNM (Buchwald and Casciotti, unpublished), (3) the role of nitrification in near-surface N_2_O production (Dore et al., 1998; Santoro et al., 2010, 2011), and (4) the role of nitrite oxidation in recycling NO^−^_3_ in and around ODZs (Sigman et al., 2005; Casciotti and McIlvin, 2007; Casciotti, 2009). Understanding the isotopic systematics for nitrification is also important for tracking the balance of high-latitude and low-latitude productivity and N budget processes (N fixation and denitrification) through NO^−^_3_ isotope distributions in the deep ocean (Sigman et al., 2009). In order to understand these applications we first review the N and O isotopic systematics of the nitrification process, including both ammonia and nitrite oxidation.

## Isotope systematics for ammonia oxidation

The δ^18^O value of NO^−^_2_ produced during ammonia oxidation (δ^18^O_NO_2_,nit_ = (^18^O/^16^O_NO_2__ ÷ ^18^O/^16^O_VSMOW_ − 1) × 1000) is dependent on the δ^18^O values of the oxygen atom sources (O_2_ and H_2_O), isotopic fractionation during their incorporation (^18^ε_k,O_2__ and ^18^ε_k,H_2_O,1_, respectively), as well as any exchange of oxygen atoms between nitrite and water (*x*_AO_) and the corresponding equilibrium isotope effect (^18^ε_eq_) (Equation 1; Casciotti et al., 2011). Throughout this review, kinetic isotope fractionation factors are defined by α_k_ = k^l^/k^h^ where k^l^ is the first order rate constant for reaction of the light isotope and k^h^ is that for reaction of the heavy isotope. Equilibrium fractionation factors are defined as α_eq_ = R_1_/R_2_ where R_1_ and R_2_ are the isotope ratios of two species in equilibrium. Kinetic and equilibrium isotope effects are defined by ε = (α − 1) × 1000.

(1) $$\delta^{18}{\text{O}}_{{\text{NO}}_{2,\text{nit}}}=\left[\frac{1}{2}\left(\delta^{18}{\text{O}}_{{\text{O}}_{2}}{-}^{18}\varepsilon_{{\text{O}}_{2}}\right)+\frac{1}{2}\left(\delta^{18}{\text{O}}_{{\text{H}}_{2}\text{O}}{-}^{18}\varepsilon_{k,\;{\text{ H}}_{2}\text{O},\;1}\right)\right]\;\times \;\left(1-x_{\text{AO}}\right)+\left(\delta^{18}{\text{O}}_{{\text{H}}_{2}\text{O}}{+}^{18}\varepsilon_{\text{eq}}\right)\left(x_{\text{AO}}\right)$$

Even though oxygen is incorporated enzymatically from O_2_ to H_2_O in a 1:1 ratio during ammonia oxidation (Andersson and Hooper, 1983), early studies of AOB found that a large amount of oxygen atom exchange with water could be associated with ammonia oxidation (Dua et al., 1979; Andersson et al., 1982; Andersson and Hooper, 1983). The conditions favoring oxygen atom exchange included high cell densities and high NO^−^_2_ concentrations. These findings, as well as the low variation of deep ocean δ^18^O_NO_3__ (Casciotti et al., 2002; Sigman et al., 2009) led researchers to assume that the O atoms in oceanic NO^−^_3_ derive primarily from H_2_O with little residual signal from dissolved O_2_. In more recent studies, however, the amount of biologically-catalyzed exchange has been determined under lower cell densities and substrate concentrations and found to be much lower for marine AOB (Casciotti et al., 2010; Buchwald et al., 2012) and AOA (Santoro et al., 2011). Exchange levels were particularly low (5%) when NO^−^_2_ concentrations were held near 1 μm by co-cultivation with NOB (Buchwald et al., 2012). These results suggested that oxygen isotope exchange during nitrification may be quite low where ammonia and nitrite oxidation are tightly coupled, but may play a role when ammonia and nitrite oxidation become decoupled, such as in the PNM.

Given low amounts of biologically-catalyzed oxygen atom exchange with H_2_O, the low δ^18^O values of NO^−^_3_ in seawater may be surprising given the high δ^18^O values of dissolved O_2_ (Kroopnick and Craig, 1976). However, oxygen atom incorporation from O_2_ and/or H_2_O during ammonia oxidation is associated with isotopic fractionation, such that the ^18^O:^16^O of oxygen atoms incorporated into NO^−^_2_ is significantly lower than the ambient pools of O_2_ and H_2_O (Casciotti et al., 2010; Santoro et al., 2011). This leads to production of NO^−^_2_ from ammonia oxidation with δ^18^O values between −3‰ and 5‰ rather than near 12‰, which would be expected from average δ^18^O_H_2_O_ and δ^18^O_O_2__ values (Casciotti et al., 2010). Furthermore, since oxygen atom exchange occurs with an equilibrium isotope effect (^18^ε_eq_) of 11–14‰ (Casciotti et al., 2007; Buchwald and Casciotti, unpublished), this equilibration would tend to raise the δ^18^O value of NO^−^_2_ relative to the initial δ^18^O_NO_2__ produced by ammonia oxidation.

Nitrogen isotopic fractionation during ammonia oxidation ranges from 14‰ to 38‰ for AOB (Mariotti et al., 1981; Yoshida, 1988; Casciotti et al., 2003) and 20–22‰ for AOA (Santoro and Casciotti, 2011). These values represent the isotope effect expressed under non-limiting concentrations of NH^+^_4_. In the ocean NH^+^_4_ consumption generally goes to completion, so the isotope effect for ammonia oxidation may not be expressed. It may, however, be expressed at the branch point between ammonia assimilation and oxidation in the euphotic zone (Wankel et al., 2007; DiFiore et al., 2009) or in the production of N_2_O by ammonia oxidizers (Yoshida, 1988; Frame and Casciotti, 2010).

## Isotope systematics for N_2_O production

**Figure 1:**
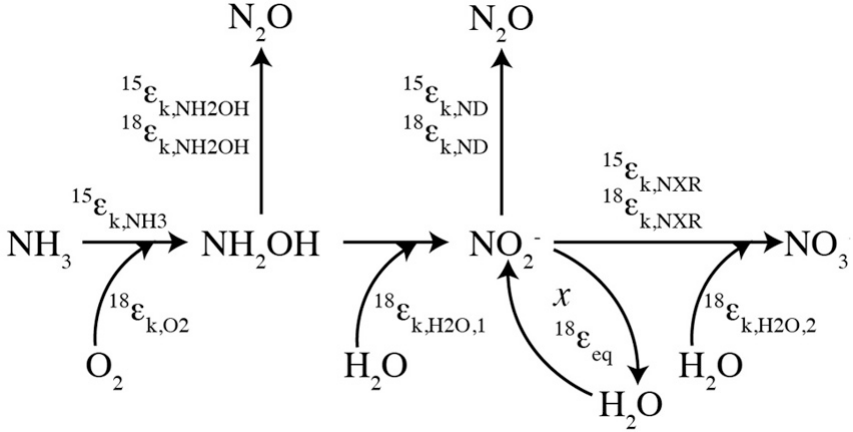
**Isotopic systematics for nitrification.** A schematic of the isotopic systematics for ammonia oxidation and nitrite oxidation during nitrification. The kinetic isotope effects for ammonia oxidation (^15^ε_k,NH3_) and nitrite oxidation (^15^ε_k,NXR_ and ^18^ε_k,NXR_) characterize the isotopic fractionation for the main N transformation processes while isotopic fractionation during oxygen atom incorporation (^18^ε_k,O_2__, ^18^ε_k,H_2_O,1_, and ^18^ε_k,H_2_O,2_) controls the oxygen isotopes incorporated by the central pathway. Oxygen isotope exchange during ammonia oxidation and/or post-production abiotic exchange (*x*) may also play a role through the equilibrium fractionation (^18^ε_eq_) associated with it. N_2_O production occurs with N and O fractionation through decomposition of hydroxylamine (NH_2_OH) and nitrifier-denitrification (ND).

**Figure 2:**
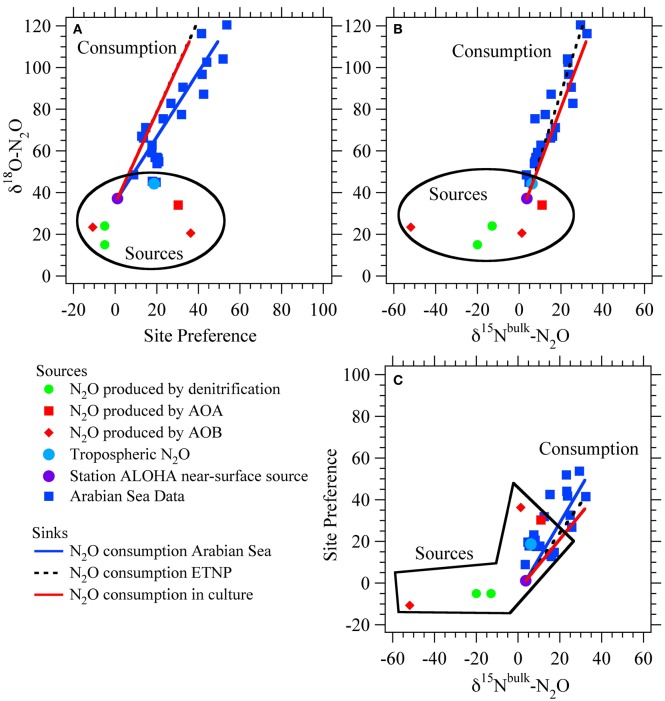
**Isotopic signatures for nitrous oxide sources and sinks.** Isotope-isotope plots for N_2_O sources from ammonia-oxidizing archaea (AOA; Santoro et al., 2011), nitrification and nitrifier-denitrification by ammonia-oxidizing bacteria (AOB; Frame and Casciotti, 2010), and production by denitrification of NO^−^_3_ or NO^−^_2_ (Barford et al., 1999; Casciotti et al., 2007). Also shown are average tropospheric air (Kim and Craig, 1990; Yoshida and Toyoda, 2000; Croteau et al., 2010) and the estimated near-surface source at Station ALOHA in the North Pacific Subtropical Gyre (Popp et al., 2002). The isotopic trends for N_2_O consumption by denitrification are based on the Arabian Sea data (McIlvin and Casciotti, 2010), ETNP data (Yamagishi et al., 2007), and culture studies (Ostrom et al., 2007). Sources and sinks are distinguished by their effects on d18O-N_2_O vs. SP **(A)**, d18O-N_2_O vs. d15Nbulk-N_2_O **(B)**, and SP vs. d15Nbulk-N_2_O **(C)**.

Production of N_2_O by AOB occurs through two separate pathways: hydroxylamine decomposition and nitrite reduction, so-called “nitrifier denitrification” (Figure 1; Poth and Focht, 1985; Hooper et al., 1990). The isotopic compositions (δ^15^N^bulk^, δ^18^O, δ^15^N^α^, δ^15^N^β^, and site preference (SP) = δ^15^Nα − δ^15^N^β^) of the N_2_O produced through these pathways may provide insight into the mechanisms of N_2_O production under different growth conditions (Frame and Casciotti, 2010; Sutka et al., 2003, 2004). For example, N_2_O production through nitrifier denitrification (enhanced by high cell densities, high NO^−^_2_ concentrations, and low O_2_ concentrations; Frame and Casciotti, 2010) has low δ^15^N^bulk^ and low SPs relative to that produced by hydroxylamine decomposition (Figure 2). This is most likely due to the additional steps involved with the production of N_2_O from NO^−^_2_ and accumulation of the main product, NO^−^_2_, which enables fractionation associated with NO^−^_2_ reduction to be expressed.

Oxygen isotopes have been underutilized in determining N_2_O sources, primarily because the isotopic systematics are less well understood, but knowledge of the O isotope systematics is increasing (Frame and Casciotti, 2010; Snider et al., 2012). The N_2_O produced via nitrifier denitrification has a slightly lower δ^18^O value than that produced from hydroxylamine decomposition (Figure 2; Frame and Casciotti, 2010). This is most likely because H_2_O is incorporated into NO^−^_2_, leading to lower δ^18^O values in NO^−^_2_ relative to NH_2_OH. However, going from either NH_2_OH or NO^−^_2_ to N_2_O involves the loss of O atoms, which can occur with fractionation. This fractionation leads to preferential loss of ^16^O and retention of ^18^O in the residual N oxides transferred to N_2_O. The net isotopic fractionation for oxygen isotopes in the hydroxylamine decomposition pathway (^18^ε_NH2OH_), including both incorporation of O_2_ into NH_2_OH and production of N_2_O from NH_2_OH, was 2.9 ± 0.8‰ indicating that N_2_O produced from this pathway had a lower ^18^O:^16^O than the ambient O_2_ (Frame and Casciotti, 2010). The net isotope effect for N_2_O production from NO^−^_2_ via nitrifier denitrification (^18^ε_ND_) was −8.4 ± 1.4‰ (Frame and Casciotti, 2010). The negative value indicates that the N_2_O produced from NO^−^_2_ is enriched in ^18^O relative to NO^−^_2_, consistent with branching of O atoms and preferential loss of ^16^O during this reaction (Casciotti et al., 2007).

The N_2_O site preference (SP) is determined mainly by the enzymatic mechanism, rather than the substrate δ^15^N value (Toyoda and Yoshida, 1999; Yoshida and Toyoda, 2000; Schmidt et al., 2004). The SP of N_2_O produced during nitrification is +30‰ to +38‰ (Figure 2; Sutka et al., 2003, 2004; Frame and Casciotti, 2010), while N_2_O produced from denitrification and nitrifier denitrification has a SP of −10‰ to +5‰ (Sutka et al., 2003, 2004; Toyoda et al., 2005; Frame and Casciotti, 2010). The large difference between the SP values of these two primary mechanisms for N_2_O production provides a large signal with which to distinguish their contributions. The interpretation of SP values is therefore somewhat simplified relative to bulk δ^15^N and δ^18^O values that reflect both mechanism and substrate isotope ratios, which change over time. This seemingly simple distinction is complicated, however, by the fact that N_2_O consumption during denitrification increases SP (Ostrom et al., 2007; Yamagishi et al., 2007; Koba et al., 2009). Therefore, a high SP value may arise through production of N_2_O via nitrification or net N_2_O consumption during denitrification. However, the δ^18^O signature of these two scenarios is quite different and can enable the scenarios to be distinguished (Figure 2).

Recently, the isotopic compositions of N_2_O produced by AOA were found to be distinct from AOB (Santoro et al., 2011). In particular, N_2_O produced by AOA is enriched in ^15^N and ^18^O relative to that produced by AOB, which may explain some of the elevated δ^15^N and δ^18^O values observed in oceanic N_2_O (Santoro et al., 2011). The reasons for the isotopic distinction between AOA and AOB is not known, but may involve a different mechanism of N_2_O production involving a unique intermediate or enzymatic pathway. However, the SP of N_2_O produced by AOA is similar to that of N_2_O produced by hydroxylamine decomposition by AOB (Santoro et al., 2011; Loescher et al., 2012). While it is not yet clear whether N_2_O production (or nitrification in general) by AOA involves hydroxylamine, isotopic evidence to date shows that the N_2_O produced aerobically by AOA does not have a SP consistent with denitrification or nitrifier-denitrification. δ^18^O data also show that the N_2_O produced by AOA incorporates O primarily from O_2_, rather than from H_2_O, which supports production by decomposition of an intermediate, rather than from NO^−^_2_ under the conditions tested (Santoro et al., 2011). It is still unknown whether AOA are able to produce N_2_O through a second pathway similar to nitrifier denitrification and thus produce N_2_O with a lower SP. Genetic analyses currently suggest that nitrification in AOA may proceed via a NO or HNO intermediate (Walker et al., 2010), which could potentially be converted to N_2_O. Further work is required to determine the pathway and intermediates of nitrification and N_2_O production by AOA, and to further study its isotope systematics under a variety of growth conditions.

## Isotope systematics for nitrite oxidation

**Figure 3:**
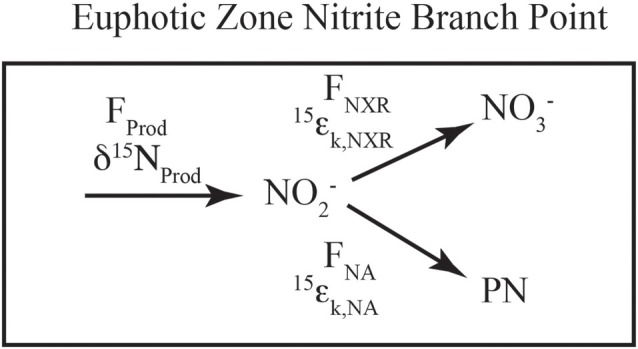
**Schematic of euphotic zone nitrite branch point.** A schematic of the fluxes and isotope effects involved with NO^−^_2_ consumption in the euphotic zone. NO^−^_2_ is produced (F_prod_, δ^15^N_prod_) from ammonia oxidation and/or nitrate reduction, the mixture of which sets the incoming flux and δ^15^N value. NO^−^_2_ consumption can occur through nitrite oxidation (F_NXR_, ^15^ε_k,NXR_) or nitrite assimilation by phytoplankton (F_NA_, ^15^ε_k,NA_). The relative rates of uptake vs. oxidation dictate the partitioning between NO^−^_2_ and NO^−^_3_ relative to the source(s) of NO^−^_2_.

The isotopic systematics for nitrite oxidation to nitrate have also been studied recently, and were found to occur with extremely unique inverse kinetic isotope effects for N (Casciotti, 2009) and O isotopes (Buchwald and Casciotti, 2010). Because of these inverse isotope effects, when nitrite oxidation is active, the δ^15^N_NO_2__ and δ^18^O_NO_2__ values are expected to be lower than the NO^−^_2_ initially produced by ammonia oxidation or nitrate reduction. As discussed below, this appears to occur in both primary and secondary nitrite maxima (Casciotti, 2009; Buchwald and Casciotti, unpublished). In most parts of the ocean, however, NO^−^_2_ does not accumulate and the isotope effects associated with nitrite oxidation can only be expressed through a branch point (Figure 3). Isotopic separation can occur at a branch point because there is more than one fate for NO^−^_2_ (e.g., NO^−^_2_ is either oxidized to NO^−^_3_ or assimilated into particulate N, PN) and the heavy isotope can be preferentially shunted in one direction vs. the other. This is analogous to the branch point that has been described during the oxidation or assimilation of ammonium (Sigman et al., 2005; Wankel et al., 2007; DiFiore et al., 2009). The equations that describe the steady state N isotopic partitioning between NO^−^_2_ and NO^−^_3_ when nitrite oxidation and assimilation occur concurrently are:

(2) $$\delta^{15}{\text{N}}_{{\text{NO}}_{2}}=\delta^{15}{\text{N}}_{{\text{NO}}_{2},\text{produced}}+{\text{f}}_{\text{NA}}\times^{15}\varepsilon_{k,\text{NA}}+{\text{f}}_{\text{NXR}}\times^{15}\varepsilon_{k,\text{NXR}}$$

(3) $$\delta^{15}{\text{N}}_{{\text{NO}}_{3},\text{produced}}=\delta^{15}{\text{N}}_{{\text{NO}}_{2}}{-}^{15}\varepsilon_{k,\text{NXR}}$$

where f_NA_ and f_NXR_ are the fractions of NO^−^_2_ consumed by assimilation and oxidation, respectively, and ^15^ε_k,NA_ and ^15^ε_k,NXR_ are the respective isotope effects. In general, nitrite oxidation will transfer NO^−^_2_ with an elevated ^15^N:^14^N ratio to the NO^−^_3_ pool, while nitrite assimilation transfers the residual NO^−^_2_ with a lower ^15^N:^14^N ratio into the PN pool. If ^15^ε_k,NA_ is 1‰ (Waser et al., 1998), ^15^ε_k,NXR_ is −15‰ (Buchwald and Casciotti, 2010), δ^15^N_NO_2__ at steady state will be lower than the source of NO^−^_2_, unless nitrite assimilation is >95% of the NO^−^_2_ sink. This has the opposite sense of the ammonia oxidation/assimilation branching where ammonia oxidation transfers low ^15^N:^14^N material into the NO^−^_2_ and NO^−^_3_ pools and higher ^15^N:^14^N material into the PN pool.

When nitrite oxidation is tightly coupled to ammonia oxidation and NO^−^_2_ does not accumulate, the δ^18^O value of the NO^−^_3_ produced primarily reflects the δ^18^O values of the O atom sources (H_2_O and O_2_; Kumar et al., 1983) and the incorporation isotope effects for ammonia and nitrite oxidation (Buchwald et al., 2012). The oxygen isotope systematics of nitrite oxidation can be described by Equation 4, while the full oxygen isotope systematics of nitrification starting from NH^+^_4_, assuming no biologically-catalyzed oxygen atom exchange during nitrite oxidation (*x*_NO_ = 0; DiSpirito and Hooper, 1986; Friedman et al., 1986; Buchwald and Casciotti, 2010), is described by Equation 5.

(4) $$\delta^{18}{\text{O}}_{{\text{NO}}_{3},\text{final}}=\frac{2}{3}\left[(1-x_{\text{NO}})\delta^{18}{\text{O}}_{{\text{NO}}_{2}}+x_{\text{NO}}\left(\delta^{18}{\text{O}}_{{\text{H}}_{2}\text{O}}{+}^{18}\varepsilon_{\text{eq}}\right)\right]+\frac{1}{3}\left(\delta^{18}{\text{O}}_{{\text{H}}_{2}\text{O}}{-}^{18}\varepsilon_{k,{\text{ H}}_{2}\text{O},\text{ 2}}\right)$$

(5) δ18ONO3,final=[23+13xAO]δ18OH2O+13[(δ18OO2−18εk, O2−18εk, H2O, 1)(1−xAO)−18εk, H2O, 2]+23ε18eq(xAO)

Equation 5 indicates that the δ^18^O_NO_3__ produced by tightly-coupled ammonia and nitrite oxidation should reflect variations in both δ^18^O_O_2__ and δ^18^O_H_2_O_ in a ratio of 1 to 2, with slight modification of this stoichiometry by biologically-catalyzed oxygen atom exchange during ammonia oxidation (Casciotti et al., 2010; Buchwald et al., 2012). As discussed below, when ammonia and nitrite oxidation are not tightly coupled, abiotic equilibration can affect δ^18^O_NO_2__ and the final δ^18^O_NO_3__ produced. Regardless of whether NO^−^_2_ accumulates, isotopic fractionation during oxygen atom incorporation should lead to an isotopic offset between the substrates (O_2_ and H_2_O) and the produced NO^−^_3_. The expected δ^18^O_NO_3__ value produced in oxygenated seawater with little exchange is −1‰ to +1‰ (similar to δ^18^O_H_2_O_), resulting from a complex series of fractionation factors rather than the unfractionated incorporation of and exchange with H_2_O (Buchwald et al., 2012).

## Abiotic equilibration of oxygen atoms in nitrite

As introduced above, abiotic equilibration of oxygen atoms between NO^−^_2_ and H_2_O is likely to play a role in setting δ^18^O_NO_2__ and δ^18^O_NO_3__ values observed in the ocean. This process does not change the concentration of NO^−^_2_ nor it's δ^15^N value, only its δ^18^O value. Oxygen atom equilibration shifts a δ^18^O_NO_2__ value from its biological starting point or “end member,” set by the isotopic systematics for biological production and consumption, toward the equilibrated δ^18^O_NO_2__ value, dictated by ambient δ^18^O_H_2_O_ and the equilibrium isotope effect for the exchange (^18^ε_eq_), which is dependent on temperature (McIlvin and Casciotti, 2006; Buchwald and Casciotti, unpublished). The relevance of abiotic exchange depends on the rates of biological turnover of nitrite relative to the rate of oxygen atom exchange with water. Where nitrite turns over quickly and does not accumulate, there is little opportunity for abiotic exchange to occur. Where nitrite turns over more slowly (several weeks-months), abiotic exchange can play an important role in δ^18^O_NO_2__ and δ^18^O_NO_3__ (Buchwald et al., 2012).

The tendency of NO^−^_2_ to exchange oxygen atoms abiotically with H_2_O at typical seawater pH and temperature conditions suggests a utility of NO^−^_2_ oxygen isotopes as a tracer for determining the rate of biological turnover of NO^−^_2_ (Buchwald and Casciotti, unpublished). This provides a unique approach to determining rates of biological processes based on static isotope measurements, without bottle incubation and associated perturbations of the system. Applications such as this move us from laboratory studies of isotope effects to a deeper understanding of the cycling of N in the environment. There are many additional examples of how knowledge of the isotope effects for nitrification has enabled advances in our understanding of the marine N cycle, and we highlight a few below.

## Implications for understanding N cycling in oxygen deficient zones

As mentioned above, processes that occur in ODZs are important for the marine N budget. Both denitrification and anammox can occur in these regions, producing N_2_ gas from dissolved inorganic nitrogen (DIN) compounds thereby removing them from the nutrient inventory. The magnitudes of these fluxes have been estimated in many different ways: through isotope tracer experiments (Kuypers et al., 2005; Thamdrup et al., 2006; Hamersley et al., 2007; Lam et al., 2009; Ward et al., 2009; Bulow et al., 2010; Jensen et al., 2011), as well as geochemical techniques based on NO^−^_3_ deficit calculations (Cline and Richards, 1972; Naqvi et al., 1982; Codispoti and Christensen, 1985; Naqvi and Sen Gupta, 1985; Gruber and Sarmiento, 1997; Deutsch et al., 2001) and biogenic N_2_ production (Devol et al., 2006; Chang et al., 2010). The ^15^N experiments in particular showcase a complex series of interacting processes cycling N in and around ODZs that can vary sporadically in space and time. What controls the overall rate of N_2_ production is not known with certainty, although it is most likely tied directly or indirectly to organic carbon supply (Ward et al., 2008). Natural abundance stable isotopes provide an integrative longer-term view of the average rates of the major fluxes of N that can be used to complement short-term incubation studies. For example, natural abundance δ^15^N_NO_3__ and δ^18^O_NO_3__ measurements have been used to estimate the relative rates of N cycle processes such as N fixation and denitrification (Brandes et al., 1998; Sigman et al., 2005).

Another aspect of N cycling in ODZs that is of great interest is the fate of NO^−^_2_ that is produced in ODZs. Once produced, NO^−^_2_ can be consumed through oxidation, regenerating NO^−^_3_, or reduction to N_2_ and loss from the nutrient inventory. Since nitrite oxidation is believed to be an oxygen requiring process, the fate of NO^−^_2_ in the oxygen deficient zone has generally been assumed to be through nitrite reduction. However, it has been shown though a variety of approaches that NO^−^_2_ can also be oxidized to NO^−^_3_ in and around ODZs. For example, early 1-D modeling studies suggested that a large fraction of NO^−^_2_ produced by nitrate reduction is reoxidized to NO^−^_3_, likely on the fringes of the oxygen deficient zone (Anderson et al., 1982). More recent nutrient profile modeling suggests that NO^−^_2_ could be oxidized to NO^−^_3_ within the oxygen deficient zone itself (Lam et al., 2011). Furthermore, direct evidence for NO^−^_2_ oxidation to NO^−^_3_ within the ODZ comes from short-term ^15^N incubation experiments (Lipschultz et al., 1990; Füssel et al., 2012).

The importance of nitrite oxidation as a sink of NO^−^_2_ in and around ODZs is supported by natural abundance isotope measurements of NO^−^_3_ and NO^−^_2_, which integrate over longer periods. Sigman et al. (2005) and Casciotti and McIlvin (2007) found that nitrite oxidation could be an important sink for NO^−^_2_ at the top of the SNM based on δ^15^N_NO_3__ and δ^18^O_NO_3__ measurements. Casciotti (2009) also showed the need for nitrite oxidation to explain the large δ^15^N differences between NO^−^_3_ and NO^−^_2_ (Δδ^15^N = δ^15^N_NO_3__ − δ^15^N_NO_2__) observed within ODZs (Casciotti and McIlvin, 2007). Although the isotope effect for NO^−^_3_ reduction to NO^−^_2_ is approximately 25‰ (Brandes et al., 1998; Voss et al., 2001), Δδ^15^N values within the SNM ranged from 25‰ to 40‰ (Casciotti and McIlvin, 2007). At steady state, Δδ^15^N is given by equation 6:

(6) $$\Delta \delta^{15}\text{N}=\delta^{15}{\text{N}}_{{\text{NO}}_{3}}-\delta^{15}{\text{N}}_{{\text{NO}}_{2}}{=}^{15}\varepsilon_{k,\text{NAR}}-{\text{F}}_{\text{NXR}}/{\text{F}}_{\text{NAR}}\times^{15}\varepsilon_{k,\text{NXR}}-{\text{F}}_{\text{NIR}}/{\text{F}}_{\text{NAR}}\times^{15}\varepsilon_{k,\text{ NIR}}$$

where F_NAR_, F_NXR_, and F_NIR_ are the fluxes from nitrate reduction, nitrite oxidation, and nitrite reduction, respectively, and ^15^ε_k,NAR_, ^15^ε_k,NXR_, and ^15^ε_k,NIR_ are the respective N isotope effects. At steady state, the large Δδ^15^N values cannot be explained by reductive processes alone since nitrite reduction would be expected to increase δ^15^N_NO_2__, thereby decreasing Δδ^15^N below 25‰. The only known mechanism for increasing Δδ^15^N above 25‰ is through NO^−^_2_ consumption with an inverse kinetic isotope effect, such as observed in nitrite oxidation (Casciotti, 2009; Buchwald and Casciotti, 2010). If all NO^−^_2_ consumption occurs through oxidation (F_NXR_/F_NAR_ = 1) with a kinetic isotope effect of −15‰, then Δδ^15^N at steady state should approach 40‰. If all NO^−^_2_ consumption occurs through nitrite reduction (F_NXR_/F_NAR_ = 0) with a kinetic isotope effect of +15‰, then Δδ^15^N would be expected to approach 10‰ at steady state. The δ^15^N difference between NO^−^_3_ and NO^−^_2_ may therefore be diagnostic of NO^−^_2_ sinks in ODZs (Casciotti, 2009).

While nitrite oxidation is generally considered to be an oxygen requiring process, O_2_ is not required as an enzymatic substrate for nitrite oxidation. Rather, O_2_ is used as an electron acceptor to support the oxidation of NO^−^_2_ to NO^−^_3_. Therefore, if an alternative electron acceptor could be substituted, nitrite oxidation may proceed in the absence of O_2_. The alternate electron acceptors that can be used by NOB for nitrite oxidation remain to be determined, but oxidation of NO^−^_2_ by species such as iodate (IO^−^_3_), Fe(III), and Mn(IV) would be thermodynamically feasible. Moreover, as mentioned above, there is independent evidence based on ^15^N incubations for nitrite oxidation occurring within the ODZs in the ETSP (Lipschultz et al., 1990) and Namibian upwelling (Füssel et al., 2012). The presence of nitrite oxidizing bacteria from the genera *Nitrospina* and *Nitrococcus* comprising up to 9% of the microbial community in the Namibian upwelling (Füssel et al., 2012) also gives strong support to their success even in low oxygen environments.

Of course, even if nitrite oxidation is occurring in ODZs, more than one process may contribute, as both bacterial nitrite oxidizers and anammox bacteria can oxidize NO^−^_2_ to NO^−^_3_. The contribution of anammox to nitrite oxidation can be estimated by comparison of F_NXR_/F_NIR_ required to explain the isotopic data with that observed during anammox (0.26:1.06; Strous et al., 2006). This ratio places an upper limit on the amount of nitrite oxidation that could be catalyzed by anammox. If the ratio of nitrite oxidation to nitrite reduction necessary to explain observed Δδ^15^N values is greater than this, then contributions from bacterial nitrite oxidation would be inferred (Casciotti, 2009). If the ratio of nitrite oxidation to nitrite reduction required to explain the isotopic data is less than this, then nitrite oxidation could potentially all be catalyzed by anammox, although denitrification may be required to explain the additional nitrite reduction. This analysis thus provides a new constraint on the relative rates of anammox and denitrification, integrated over long time periods. However, it assumes that the isotope effects for anammox are similar to denitrification for nitrite reduction and similar to nitrite oxidation for that step. Thus, the approach can be refined with additional information about the isotopic systematics of anammox.

## Implications for understanding NO^−^_3_ cycling and budgets: Δ(15, 18) revisited

**Figure 4:**
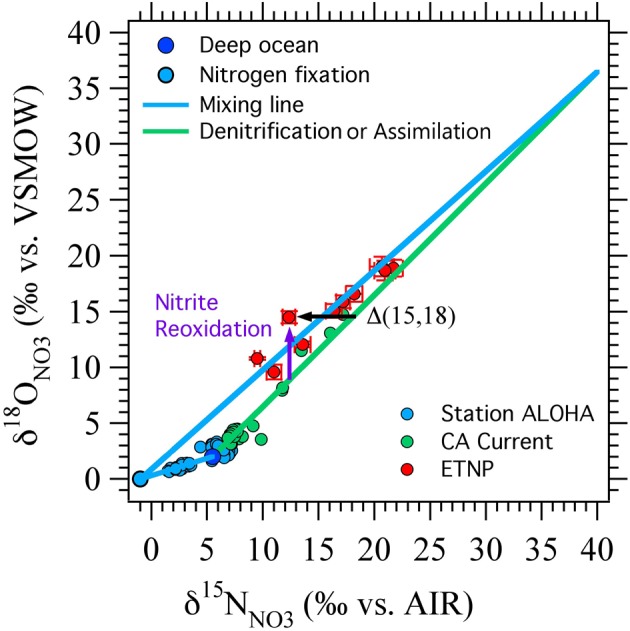
**Δ(15, 18) as originally devised.** A schematic showing the effects of nitrate reduction, assimilation, and input of NO^−^_3_ from nitrogen fixation linked to nitrification on δ^15^N_NO_3__, δ^18^O_NO_3__, and the nitrate isotope anomaly, Δ(15, 18) (black arrow). Deep ocean nitrate (dark blue circle) starts with δ^15^N_NO_3__ of 5‰ and δ^18^O_NO_3__ of 2‰. Nitrate assimilation and denitrification increase δ^15^N and δ^18^O in a 1:1 ratio ((Granger et al., 2004, 2008, 2010); green line). Remineralization of newly fixed N is assumed to add NO^−^_3_ with δ^15^N_NO_3__ of −1‰ and δ^18^O_NO_3__ of 0‰ (light blue circle, blue mixing lines). Nitrite reoxidation is expected to generally increase δ^18^O_NO_3__ relative to δ^15^N_NO_3__ because of the oxygen isotope systematics of nitrate reduction and nitrite oxidation (purple arrow). Data from station ALOHA (Casciotti et al., 2008), California Current (Santoro et al., 2010) and ETNP (Casciotti and McIlvin, 2007) are shown for comparison.

Knowing the isotopic systematics of nitrification is critical for interpreting δ^18^O_NO_3__, δ^18^O_NO_2__, and δ^18^O_N2O_ measurements from the ocean. The culture studies described above have advanced our understanding of the oxygen isotope systematics of nitrification; however, there are also constraints from field data (Casciotti et al., 2002; Sigman et al., 2009). Casciotti et al. (2002) used the nitrate δ^18^O data to put the first constraints on the δ^18^O value of NO^−^_3_ produced in the ocean. These estimates showed that NO^−^_3_ is most likely produced with δ^18^O values close to those of seawater (0‰) and were used by Sigman et al. (2005) to constrain the rates of N_2_ fixation and nitrite reoxidation from δ^15^N_NO__3_ to δ^18^O_NO__3_ data. In order to do this, Sigman et al. (2005) introduced a NO^−^_3_ isotope anomaly based on expected enrichments of δ^15^N_NO_3__ and δ^18^O_NO_3__ due to nitrate assimilation or nitrate reduction during denitrification:

(7) $$\Delta (15,\;18)=\left(\delta^{15}{\text{N}}_{{\text{NO}}_{3}}-\delta^{15}{\text{N}}_{{\text{NO}}_{3},\text{deep}}\right){-}^{18}5\varepsilon_{k,\text{NAR}}{\text{/}}^{15}8\varepsilon_{k,\text{NAR}}\times \left(\delta^{18}{\text{O}}_{{\text{NO}}_{3}}-\delta^{18}{\text{O}}_{{\text{NO}}_{3},\text{deep}}\right)$$

where δ^15^N_NO_3__ and δ^18^O_NO_3__ are the measured isotopic values of the sample, δ^15^N_NO_3_,deep_ and δ^18^O_NO_3_,deep_ are the isotopic values of unaltered deep seawater, which define the starting point for fractionation. ^18^ε_k,NAR_ and ^15^ε_k,NAR_ are the isotope effects for O and N isotopes, respectively, during nitrate reduction. While there is a wide range in the absolute values of ^18^ε_k,NAR_ and ^15^ε_k,NAR_, their ratio is very close to 1 (Granger et al., 2004, 2008, 2010). Therefore, NO^−^_3_ consuming processes generally lead to δ^15^N_NO_3__ and δ^18^O_NO_3__ values that fall along a 1:1 line and produce samples with Δ(15, 18) = 0‰ (Figure 4). Non-zero Δ(15, 18) values correspond to an enrichment of δ^18^O_NO_3__ relative to δ^15^N_NO_3__, or a depletion in δ^15^N_NO_3__ relative to δ^18^O_NO_3__, generally arising from production of NO^−^_3_ with anomalous isotopic signatures. The most likely cause for depletion in δ^15^N, especially in the nitracline of oligogrophic ocean provinces, is through remineralization of newly fixed N with a δ^15^N value near −1‰ (Capone et al., 1997; Karl et al., 1997; Meador et al., 2007). The particulate organic N produced by N fixation is remineralized to NO^−^_3_ in the subsurface, gaining O atoms from nitrification, the same process that sets the oxygen isotopic signature of NO^−^_3_ produced from other N sources. In scenario, the magnitude of Δ(15, 18) would be proportional to the N fixation flux (Sigman et al., 2005).

A relative enrichment in ^18^O, especially in the vicinity of oceanic ODZs, could represent the cycling of NO^−^_3_ through the reduction/reoxidation cycle, where the NO^−^_3_ consumed by denitrification has a similar δ^15^N_NO_3__ but a lower δ^18^O_NO_3__ value than that returned to the NO^−^_3_ pool from nitrite oxidation (Sigman et al., 2005). This formulation was successful at simulating data from regions of the ETNP where NO^−^_2_ did not accumulate (Sigman et al., 2005) and where NO^−^_2_ goes to zero at the top of the SNM (Casciotti and McIlvin, 2007). However, where NO^−^_2_ accumulates, its isotopic composition can vary dramatically within the oxygen deficient zone itself (Casciotti and McIlvin, 2007), and an interpretation including NO^−^_2_ isotope constraints is needed. The relationship between ^18^O enrichment in NO^−^_3_ and the magnitude of the nitrite reoxidation flux depends critically on the N and O isotope systematics of nitrite oxidation, which we reviewed above. Here we revisit the implications of this new knowledge for interpretations of Δ(15, 18) in euphotic zone and oxygen deficient zones.

**Figure 5:**
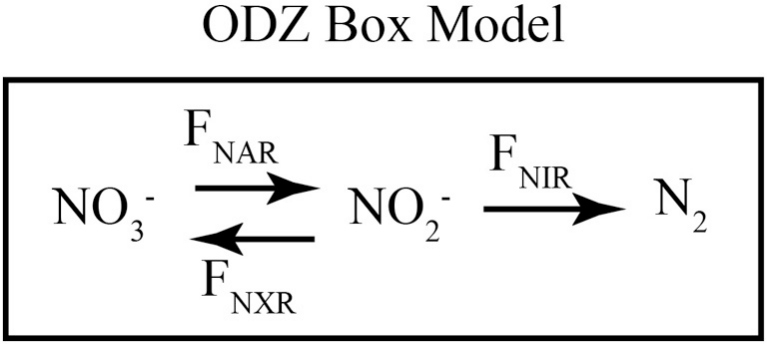
**Schematic of ODZ box model.** A schematic of the fluxes included in the time-dependent 1-box ODZ model. NO^−^_3_ is reduced to NO^−^_2_ through nitrate reduction (F_NAR_). NO^−^_2_ can be consumed either through dissimilatory nitrite reduction (F_NIR_) or nitrite oxidation (F_NXR_). The rates of these processes are assumed to be first order in NO^−^_3_ or NO^−^_2_, respectively, and isotope effects control the relative reaction of heavy and light isotopes. Table 1 gives the values of the parameters used in the model.

Using a simple time-dependent 1-box model of the ODZ N cycle, we have reevaluated the impact of nitrite reoxdiation on δ^15^N_NO_3__ and δ^18^O_NO_3__ in a hypothetical ODZ (Figure 5) and show that nitrite oxidation can either raise or lower Δ(15, 18), depending on the relative δ^15^N and δ^18^O values of NO^−^_2_ and NO^−^_3_. Our model focuses on determining the relative rates of NO^−^_2_ reoxidation to NO^−^_3_ (F_NXR_) and reduction (to NO or NH^+^_4_; F_NIR_) from NO^−^_3_ and NO^−^_2_ isotopic data. The oxidative flux is assumed to have the N and O isotopic systematics of bacterial nitrite oxidation (Buchwald and Casciotti, 2010; Table 1), regardless of whether it is carried out by bacterial nitrite oxidizers or anammox bacteria, or some mixture of the two. The reductive processes are assumed to have ^15^ε = ^18^ε = 15‰ (Table 1) regardless of whether NO^−^_2_ is reduced to N_2_ (via anammox or denitrification) or NH^+^_4_ [via denitrification to ammonium (DNRA)]. Unfortunately, very little information is currently available on the N isotope effects for nitrite reduction by these processes (Bryan et al., 1983) and no information is available for the O isotope effects. In the absence of more specific information, we make the simplifying assumption that the different nitrite reductase enzymes have similar N and O isotope effects. Clearly, this is an important area of future research.

**Table 1:** **Parameters used in oxygen deficient zone box model**.

**Parameter**	**Description**	**Value**	**Reference**
δ^15^N_NO_3_,initial_	Initial nitrate δ^15^N	5‰	Sigman et al., 2000
δ^18^O_NO_3_,initial_	Initial nitrate δ^18^O	2‰	Casciotti et al., 2002
δ^18^O_H_2_O_	Water δ^18^O value	0‰	Craig and Gordon, 1965
k_NAR_	First order rate constant for nitrate reduction	0.001 day^−1^	Estimated to achieve a rate of 20 nM day^−1^; Lam et al., 2011
k_NXR_	First order rate constant for nitrite oxidation	0–0.003 day^−1^	Estimated to achieve range of observed nitrite oxidation rates; Füssel et al., 2012; Lipschultz et al., 1990
k_NIR_	First order rate constant for nitrite reduction	0.001 day^−1^	Estimated to achieve a rate of 5 nM day^−1^; Devol et al., 2006
k_EXCH_	First order rate constant for nitrite/water exchange	0.01 day^−1^	Buchwald and Casciotti
^15^α_k,NAR_	N isotope effect for nitrate reduction	1.019	Deutsch et al., 2004; Granger et al., 2008
^15^α_k,NXR_	N isotope effect for nitrite oxidation	0.985	Casciotti, 2009; Buchwald and Casciotti, 2010
^15^α_k,NIR_	N isotope effect for nitrite reduction	1.015	Bryan et al., 1983
^18^α_NAR_	O isotope effect for nitrate reduction	1.019	Granger et al., 2008
^18^α_k,NXR_	O isotope effect for nitrite oxidation	0.997	Buchwald and Casciotti, 2010
^18^α_k,NIR_	O isotope effect for nitrite reduction	1.015	Sigman et al., 2005
^18^α_kH_2_O,2_	O isotope effect for H_2_O incorporation	1.010	Buchwald and Casciotti, 2010
^18^α_B_	Branching O isotope effect during nitrate reduction	0.975	Casciotti et al., 2007
^18^α_eq_	Equilibrium isotope effect for nitrite/water O exchange	1.014	Casciotti et al., 2007; (Buchwald and Casciotti, unpublished)

**Figure 6:**
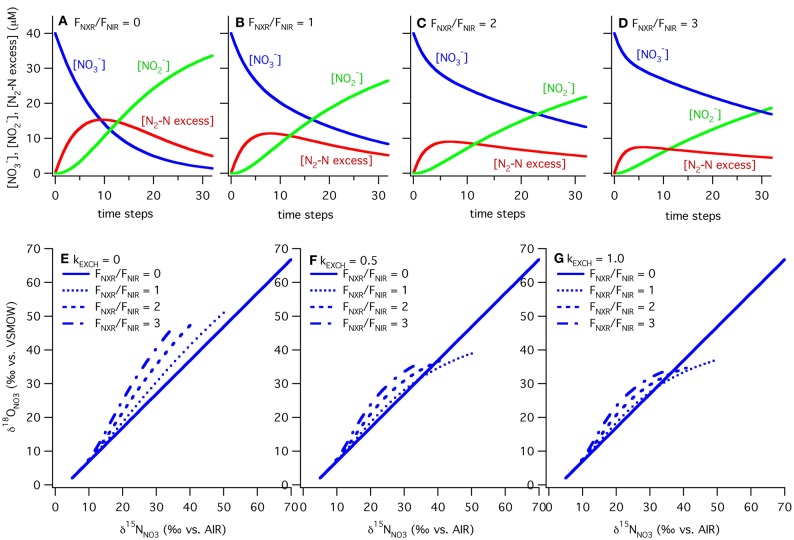
**Results of ODZ model for varying ratios of nitrite oxidation to nitrite reduction and rates of exchange.** Results from the ODZ box model at different relative rates of nitrite oxidation and nitrite reduction (F_NXR_/F_NIR_), ranging from 0 to 3. Mass balance is maintained in the model between NO^−^_3_, NO^−^_2_ and excess N_2_-N with F_NXR_/F_NIR_ = 0 (panel **A**), 1 (panel **B**), 2 (panel **C**) and 3 (panel **D**). NO^−^_2_ accumulation and N_2_ production decrease as F_NXR_ increases. The ODZ box model shows that NO^−^_2_ cycling can generate both positive and negative Δ(15, 18) values, depending on the extent of NO^−^_3_ consumption (increasing δ^15^N, δ^18^O values), the relative rates of nitrite oxidation and reduction (F_NXR_/F_NIR_), and the rate of oxygen atom exchange between NO^−^_2_ and H_2_O (k_EXCH_). In each case the slope of δ^18^O_NO_3__ vs. δ^15^N_NO_3__ is equal to 1 when F_NXR_ = 0. As F_NXR_/F_NIR_ increases, the magnitude of the Δ(15, 18) anomaly increases at a given δ^15^N value. As NO^−^_2_/H_2_O exchange increases (=0 in panel **E**, 0.5 in panel **F**, and 1.0 in panel **G**), the non-zero levels of nitrite oxidation generate positive Δ(15, 18) values, most likely due to the relative δ^18^O values of NO^−^_3_ produced and consumed under these scenarios. All parameters used in the model are reported in Table 1.

In our model, the processes are all represented as first order, and the rate constants (k's) are given in units of day^−1^ to match measured rates of nitrate reduction, nitrite reduction, and nitrite oxidation in ODZs (Table 1). The isotope effects taken from the literature are also given in Table 1. We vary the relative rates of nitrite oxidation and nitrite reduction (F_NXR_/F_NIR_) between 0 and 3 (F_NXR_ representing 0–75% of NO^−^_2_ consumption) and the rate constant for exchange (k_EXCH_) between 0 and 1 day^−1^ to evaluate the effects of changes in these parameters on simulated δ^15^N_NO_3__ and δ^18^O_NO_3__ (Figure 6). Maximum rate constants of exchange between NO^−^_2_ and H_2_O of 1 day^−1^ appear reasonable based on recent laboratory studies (Casciotti et al., 2007; Buchwald and Casciotti, unpublished). As F_NXR_/F_NIR_ increases from 0 to 3, the amount of NO^−^_3_ retained in the system increases despite an unchanging rate constant for nitrate reduction. In fact, because the reaction is taken as first order, the higher concentrations of NO^−^_3_ brought about by higher levels of F_NXR_ lead to higher overall rates of nitrate reduction. However, it is clear from the mass balances in the different scenarios that nitrite reoxidation helps buffer against excessive loss of NO^−^_3_, accumulation of NO^−^_2_, and production of N_2_ (Figures 6A–D), and may help explain why NO^−^_3_ is never fully removed in oceanic ODZs.

The magnitude of nitrite oxidation also affects the δ^15^N_NO_3__ and δ^18^O_NO_3__ patterns. When F_NXR_/F_NIR_ = 0, the δ^15^N_NO_3__ and δ^18^O_NO_3__ data fall along the 1:1 line prescribed by the isotope effects for nitrate reduction (Figures 6E–G). As F_NXR_/F_NIR_ increases, increasingly negative Δ(15, 18) values are produced. The strength of this effect is also dependent on the rate of abiotic NO^−^_2_/H_2_O exchange, with higher exchange rates partly diluting this effect and actually leading to positive Δ(15, 18) values at high extents of NO^−^_3_ consumption (the highest δ^15^N_NO_3__ values; Figure 6). This interesting phenomenon is most likely due to reversal of the impact of nitrite reoxidation on δ^18^O_NO_3__ at high δ^18^O_NO_3__ values, with nitrite oxidation returning NO^−^_3_ with a lower δ^18^O_NO_3__ value than that removed by nitrite reduction. This would be exacerbated at high rates of exchange, which helps to maintain δ^18^O_NO_2__ values at a constant level regardless of δ^18^O_NO_3__. Tuning the model to match observed δ^18^O_NO_2__ data requires a high rate of exchange relative to biological fluxes, and therefore most closely follows the k_EXCH_ = 1 scenario.

Larger ratios of F_NXR_/F_NIR_ could be imagined, but the model results from such simulations produce unrealistic Δ(15, 18) anomalies at a given δ^15^N_NO_3__ value. Furthermore, because excess N_2_ does accumulate in ODZs, we know that some NO^−^_2_ is ultimately reduced to N_2_. Indeed, we could potentially use the stoichiometry of N_2_ production in ODZs to interrogate the importance of nitrite oxidation. If nitrite oxidation is not important, the standard stoichiometry (Richards, 1965; Devol et al., 2006) of 106 CO_2_: 55.2 N_2_ would be expected, whereas higher amounts of CO_2_ would be expected if a significant fraction of the produced NO^−^_2_ is reoxidized to NO^−^_3_. This may seem counterintuitive because autotrophic nitrite oxidation should fix CO_2_ back into organic matter, but the excess NO^−^_3_ reduction required to supply the NO^−^_2_ in the first place should far outweigh the CO_2_ fixed by nitrite oxidation.

**Figure 7:**
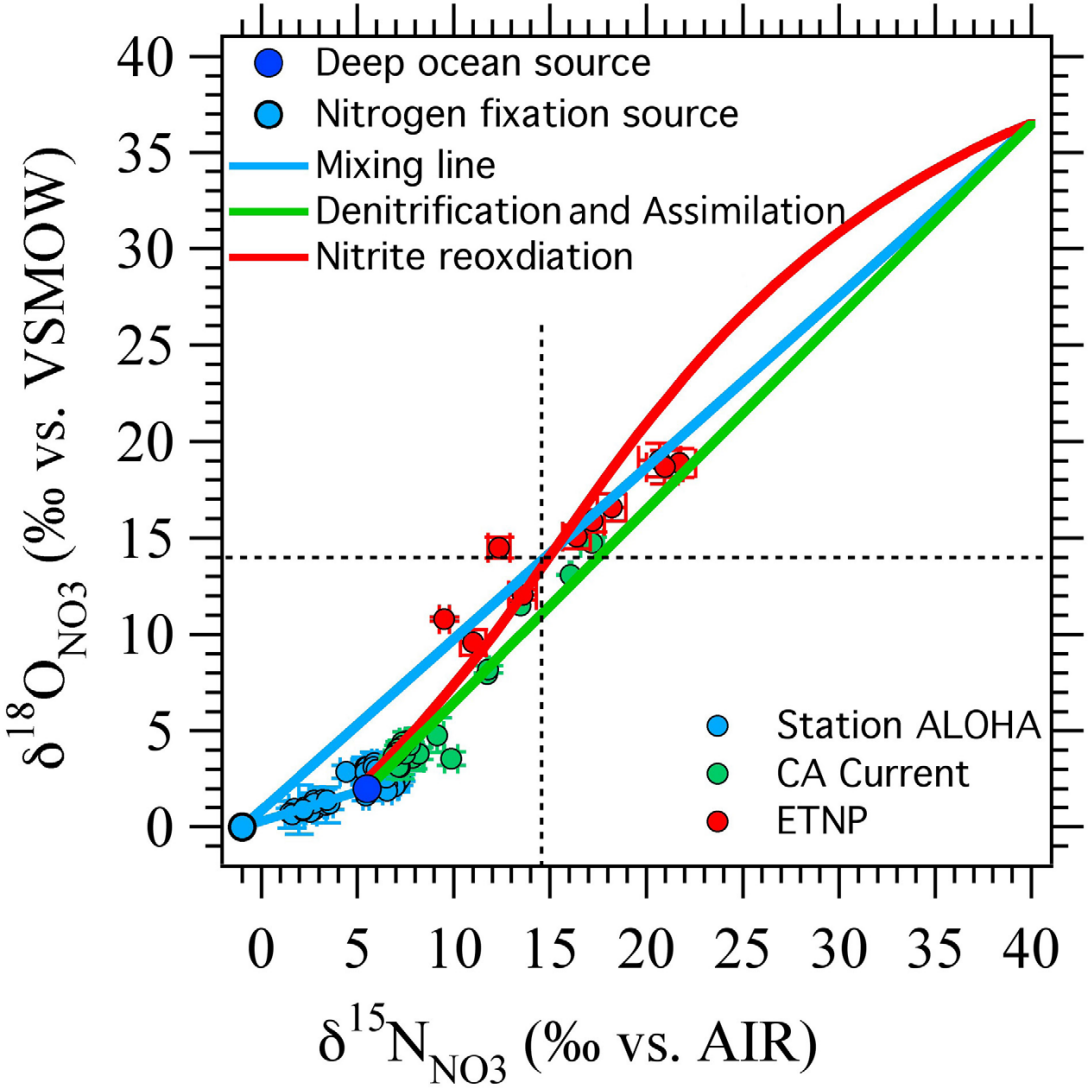
**Results from ODZ model compared against published data.** Nitrogen fixation (blue circle and blue lines) is efficient at generating negative Δ(15, 18) values at the lower range of δ^15^N_NO_3__ and δ^18^O_NO_3__ values, while nitrite reoxidation (red line) has a stronger effect at higher δ^15^N_NO_3__ and δ^18^O_NO_3__ values. The nitrite reoxidation curve shown here was generated from the box model with k_EXCH_ = 0.5 and F_NXR_/F_NIR_ = 2 (Figure 6F, dashed line). Data from station ALOHA (Casciotti et al., 2008) in the north Pacific subtropical gyre is well explained by an input from N_2_ fixation. Data from the California Current (Santoro et al., 2010) falls close to the 1:1 line suggesting little influence of nitrogen fixation or nitrite oxidation in the euphotic zone. Most of the data from the ETNP (Casciotti and McIlvin, 2007) could be explained by either nitrite reoxiation or nitrogen fixation, but two points (which fall in the shallow oxycline at the top of the SNM) require inputs from both nitrite reoxidation and nitrogen fixation.

It is interesting to note that the two scenarios for producing negative Δ(15, 18) values (N_2_ fixation and nitrite reoxidation) are each more effective at different points in NO^−^_3_ isotope space (Figure 7). N_2_ fixation is most effective at generating negative Δ(15, 18) signals at δ^15^N_NO_3__ and δ^18^O_NO_3__ values less than 10‰, near the base of the euphotic zone. In contrast, nitrite reoxidation is most effective at generating negative Δ(15, 18) signals at intermediate δ^15^N_NO_3__ and δ^18^O_NO_3__ values and extents of NO^−^_3_ consumption by denitrification, where N_2_ fixation has relatively little effect on the Δ(15, 18). Therefore, we may be able to distinguish between the processes responsible for Δ(15, 18) generation by where the anomaly lies in δ^15^N_NO_3__ vs. δ^18^O_NO_3__ space, as well as from other water column indicators. For example, using a steady state model, Casciotti and McIlvin (2007) showed that the NO^−^_3_ isotope anomaly at the top of the SNM could not be generated by N_2_ fixation alone and was consistent with oxidation of NO^−^_2_ leaking out of the top of the SNM. However, they suggested that a combination of N_2_ fixation and nitrite reoxidation may best fit the observations. This conclusion is echoed here where it is difficult to generate large Δ(15, 18) signals at these δ^15^N_NO_3__ and δ^18^O_NO_3__ values through either N_2_ fixation or nitrite reoxidation alone (Figure 7).

In addition to oxygen deficient zone and near-surface processes, NO^−^_3_ isotopes have also been used to examine the global ocean cycle and budget of NO^−^_3_ in the ocean interior (Sigman et al., 2009). This was done using an 18-box model of the global ocean where the implications of different assumptions about the oxygen isotopic systematics of nitrification could be tested. Their model was also used to constrain the relative rates of the internal N cycle (NO^−^_3_ uptake, export, and nitrification) and N budget processes (N_2_ fixation and denitrification) and the ratio of low latitude productivity, where nutrient consumption goes to completion, to high latitude productivity, where nutrient uptake is incomplete. By comparing model results to δ^15^N_NO_3__ and δ^18^O_NO_3__ data from a variety of oceanographic profiles representing the major ocean basins, the impacts of partial NO^−^_3_ assimilation in polar regions on the N and O isotopes of NO^−^_3_ in the ocean interior, and of low latitude productivity on the ^18^O enrichment in preformed NO^−^_3_ was diagnosed. N budget processes (N_2_ fixation and denitrification) led to variations in subsurface δ^15^N_NO_3__ and δ^18^O_NO_3__, but in their absence, the large scale steady state δ^18^O value of subsurface NO^−^_3_ was set by nitrate assimilation in polar regions. Nitrate uptake in the southern ocean leads to heavy isotope enrichment in preformed NO^−^_3_, while nitrate assimilation in low latitudes removes the δ^18^O signal of the preformed NO^−^_3_ and replaces it with the nitrification signal (Sigman et al., 2009). Overall, when only internal processes were active in the model, the mean ocean δ^18^O_NO_3__ value was 1.1‰ higher than the nitrification source. When the N budget was added to the model, the mean ocean δ^18^O_NO_3__ value was 2.4‰ higher than the nitrification source value. This analysis provides additional constraints on the δ^18^O value of newly produced NO^−^_3_ in the ocean to fall between −1‰ and +1‰ (Sigman et al., 2009), which is consistent with culture studies that illustrate how these values are controlled biochemically (Buchwald et al., 2012).

## Nitrogen cycling in the euphotic zone

Several studies have now used N and O isotope ratio measurements to study the relative rates of N cycling in the euphotic zone. In particular, knowledge of the isotopic systematics of nitrate uptake (Granger et al., 2004, 2010) and nitrification (Buchwald and Casciotti, 2010; Casciotti et al., 2010, 2011; Buchwald et al., 2012) enables the assessment of the relative rates of nitrification and nitrate uptake from euphotic zone NO^−^_3_ isotope data.

Wankel et al. (2007) used a steady-state box model to interpret the amount of nitrification contributing to nitrate uptake by phytoplankton in Monterey Bay, CA using δ^15^N_NO_3__ and δ^18^O_NO_3__ variations. Assuming that nitrate assimilation leads to equivalent fractionation of N and O isotopes (Granger et al., 2004), and that δ^18^O_ntr_ = 2.9‰, they estimated that nitrification could supply up to 30% of NO^−^_3_ assimilated by phytoplankton in Monterey Bay, consistent with intensive isotope tracer incubation studies (Ward, 2005). Because δ^18^O_ntr_ was uncertain at that time, they performed sensitivity studies to address the impact of different δ^18^O_ntr_ values on their interpretation. We now believe that δ^18^O_ntr_ is between −1‰ and +1‰ (Buchwald et al., 2012), and applying this to the model from Wankel et al. (2007), leads to a smaller increase in δ^18^O_NO_3__ for the same amount of nitrification. Thus, to achieve the same δ^18^O_NO_3__ enrichment in their model requires more nitrification than originally estimated.

DiFiore and colleagues (2009) estimated the amount of nitrification contributing to nitrate uptake in the euphotic zone of the Polar Antarctic Zone using a time-dependent 1-box model. Like Wankel et al. (2007), they assumed that ^18^ε_NR_ = ^15^ε_NR_ for nitrate uptake and allowed branching of NH^+^_4_ (and NO^−^_2_) between nitrification and assimilation to partition isotopes between the NO^−^_3_ and particulate N pools. One important difference from the Wankel et al. (2007) model is that they assumed δ^18^O_ntr_ = +1.1‰ based on more recent constraints on this value (Sigman et al., 2009). They inferred that δ^15^N_NO_3__ should be lowered slightly due to nitrification (offsetting the isotopic fractionation during uptake) and δ^18^O_NO_3__ should be raised (because the δ^18^O of newly produced NO^−^_3_ was higher than that removed). Both of these factors should lead to negative Δ(15, 18) values, as discussed above, but they found that nitrification had a relatively small impact on δ^15^N_NO_3__ and δ^18^O_NO_3__ values in the Polar Antarctic Zone. They concluded that in the Polar Antarctic Zone less than 1% of NO^−^_3_ assimilated by phytoplankton is likely to have been produced by nitrification in the euphotic zone (DiFiore et al., 2009). This is consistent with other estimates from the southern ocean (Olson, 1981b; Bianchi et al., 1997; Law and Ling, 2001) and quite a bit lower than other regions (Yool et al., 2007; Wankel et al., 2007; Clark et al., 2008). This elegant study provides an excellent example of how NO^−^_3_ isotopes can be used to constrain N cycle processes in an appropriate model framework.

NO^−^_3_ and NO^−^_2_ isotopes have also been used to understand the sources and cycling of NO^−^_2_ in the PNM at the base of the euphotic zone. Mackey et al. (2011) used natural abundance NO^−^_3_ + NO^−^_2_ isotope data and isotope tracer experiments to determine the sources of NO^−^_2_ to the PNM in the Gulf of Aqaba. They found active nutrient regeneration and nitrification throughout the water column. In the transition from well mixed to stratified conditions, NO^−^_2_ was generated by incomplete NO^−^_3_ reduction by light-limited phytoplankton creating a broad band of NO^−^_2_. After stratification was established, NO^−^_2_ generation by ammonia oxidation contributed to maintenance of the PNM. In both cases, NO^−^_2_ was consumed by nitrite oxidation below the PNM. Once again, nitrification was interpreted to play an important role in NO^−^_3_ isotope dynamics in the upper water column where increases in δ^18^O_NO_3__ were much higher than increases in δ^15^N_NO_3__.

In another recent study of PNM dynamics, natural abundance δ^18^O_NO_2__ and δ^15^N_NO_2__ values were used to infer the sources and average age of NO^−^_2_ in the PNM of the Arabian Sea (Buchwald and Casciotti, unpublished). Because the δ^15^N_NO_2__ and δ^18^O_NO_2__ values produced from ammonia oxidation and nitrate reduction are distinct, the sources can be readily distinguished. Based on natural abundance δ^15^N_NO_2__ and δ^18^O_NO_2__ data, ammonia oxidation was inferred to be the main source of NO^−^_2_ to the PNM in the Arabian Sea.

## Implications for interpreting N_2_O sources

Uncertainty in the isotopic composition of N_2_O produced during ammonia oxidation has hampered the interpretation of near-surface N_2_O production rates and fluxes using two-component end member models (Dore et al., 1998; Popp et al., 2002; Santoro et al., 2010). Better understanding of the oxygen isotopic systematics of nitrification can provide further insight into outstanding questions in N_2_O oxygen isotope variations, such as why δ^18^O_N2O_ in seawater is so high (Ostrom et al., 2000; Popp et al., 2002), what mechanisms of N_2_O production operate in oxyclines surrounding oceanic ODZs (Codispoti and Christensen, 1985), and what the mechanisms and controls on N_2_O production are in the near-surface ocean (Dore et al., 1998; Popp et al., 2002; Santoro et al., 2011).

For example, N_2_O production in the near-surface ocean is largely believed to be the result of nitrification. However, the isotopic composition of N_2_O in the near surface and the inferred near surface source (Dore et al., 1998) have higher δ^15^N and δ^18^O values than are characterized by bacterial ammonia oxidation (Yoshida, 1988; Frame and Casciotti, 2010). Recent evidence suggests that AOA are important for nitrification in such environments (Wuchter et al., 2006; Beman et al., 2008; Mincer et al., 2007; Church et al., 2010; Santoro et al., 2010) and that they produce N_2_O with bulk δ^15^N and δ^18^O values similar to the near-surface source (Santoro et al., 2011). These data support a role for them in near-surface N_2_O production. As discussed above, the mechanisms of N_2_O production by AOA are currently unknown, and more work is needed to characterize the N_2_O production and isotopic composition of marine AOA under a variety of growth conditions. For example, the SP of N_2_O produced by AOB varies widely with dissolved oxygen levels (Frame and Casciotti, 2010) but so far the isotopic composition of N_2_O produced by AOA has only been examined under aerobic growth conditions (Santoro et al., 2011; Loescher et al., 2012). Therefore, we do not know whether they are capable of producing N_2_O with a SP similar to near surface N_2_O (Popp et al., 2002).

## Concluding remarks

Understanding the nitrogen and oxygen isotopic systematics of nitrification can contribute greatly to our understanding of nitrogen cycling in the ocean, as nitrification is involved with transformations between the major pools of DIN (NH^+^_4_, NO^−^_2_, NO^−^_3_, and N_2_O). Both ammonia and nitrite oxidation are involved with large and distinctive isotope effects, leading to predictable patterns in the isotope ratios of compounds that they transform. The discovery of AOA and their importance in ocean biogeochemistry necessitates renewed study of the isotopic systematics of nitrification. In preliminary studies, the isotopic systematics of AOA appear similar to AOB for N isotope fractionation and O atom incorporation into NO^−^_2_ (Santoro and Casciotti, 2011; Santoro et al., 2011). However, the production of N_2_O and the isotopic systematics of this process need to be further investigated.

### Conflict of interest statement

The authors declare that the research was conducted in the absence of any commercial or financial relationships that could be construed as a potential conflict of interest.
